# A case of an elderly patient with high-grade colorectal cancer in poor general condition who showed near complete response to chemotherapy and achieved long-term survival

**DOI:** 10.1016/j.ijscr.2019.03.015

**Published:** 2019-03-22

**Authors:** Yoshiaki Kanemoto, Giichiro Tsurita, Tomohiro Kurokawa, Yuki Azuma, Kentaro Yazawa, Yoshinori Murakami

**Affiliations:** a Department of Surgery, IMSUT Hospital, The Institute of Medical Science, The University of Tokyo, Japan; b Division of Molecular Pathology, The Institute of Medical Science, The University of Tokyo, Japan

**Keywords:** PS, performance status, mFOLFOX6, 5-fluorouracil leucovorin, and oxaliplatin, Pmab, panitumumab, CR, complete response, BSC, best supportive care, ECOG, Eastern Cooperative Oncology Group, OS, overall survival, Elderly, Poor PS, mCRC, mFOLFOX6, Pmab

## Abstract

**Introduction:**

Chemotherapy is difficult to administer in patients with poor performance status (PS), advanced metastatic lesion, and unresectable colon cancer. We report herein our experience of a patient who showed complete response to chemotherapy and marked PS improvement. The patient presented with the following adverse factors poor PS, advanced progression of metastatic lesions, advanced unresectable colorectal cancer with severe stricture, and old age.

**Presentation of case:**

The patient was an 80-year-old male diagnosed with occlusive cancer of the descending colon with multiple metastases in the liver, Stage Ⅳb (National Comprehensive Cancer Network guidelines version 2. 2018). A 5-fluorouracil, leucovorin, and oxaliplatin (mFOLFOX6) + panitumumab (Pmab) regimen was successfully administered and led to decreased tumor marker levels; oral intake also became possible. Additional examinations showed that the primary lesion and distant metastatic lesions had almost disappeared; the patient had achieved a near complete response (CR). Currently, 35 cycles of mFOLFOX6＋Pmab have been administered, and his near CR has been maintained for 32 months.

**Discussion:**

Best supportive care (BSC) is the recommended option for elderly patients with advanced unresectable colon cancer. This is the first case in which an elderly patient with poor PS and advanced unresectable colorectal cancer was treated with combination chemotherapy of mFOLFOX6 + Pmab.

**Conclusion:**

Although the use of chemotherapy for elderly with advanced unresectable colorectal cancer or those with poor PS is limited, this case shows that systemic chemotherapy is now an option for such cases previously managed with BSC.

## Highlights

• Systemic therapy can achieve good treatment outcomes in advanced CRC.
• Suitable chemotherapeutics can markedly improve the prognosis of unresectable CRC.
• Unresectable CRC can now be treated with systemic chemotherapy instead of BSC.

## 1 Introduction

Initiation of chemotherapy is difficult in patients with poor performance status (PS), advanced metastatic lesion, and unresectable colon cancer, and best supportive care (BSC) is the standard treatment of choice in these patients. However, advancements in anti-cancer drugs have been remarkable, and several case reports have demonstrated that if anti-cancer drugs are properly selected and complications are prevented, chemotherapeutic drugs may have substantial effects [1].

This is a report of a patient with many adverse factors: old age, advanced unresectable colon cancer with poor PS, and advanced liver metastases.

The work in this case has been reported in line with the SCARE criteria [2].

## 2 Presentation of case

An 80-year-old man presented with complaints of general malaise, loss of appetite, and weight loss. A full physical examination revealed occlusive cancer of the descending colon with liver metastasis. Two hospitals recommended BSC. However, the patient had a strong desire to undergo anti-cancer treatment.

Upon admission, he weighed 47.9 kg, had a height of 170 cm, and had an Eastern Cooperative Oncology Group (ECOG) PS 3. The alkaline phosphatase (937 U/L), lactate dehydrogenase (1190 U/L), and γ-glutamyltransferase (494 U/L) levels were so elevated. Carcinoembryonic antigen (921 ng/mL) and CA19-9 (32,963 U/mL) tumor markers were also elevated.

**Fig. 1:**
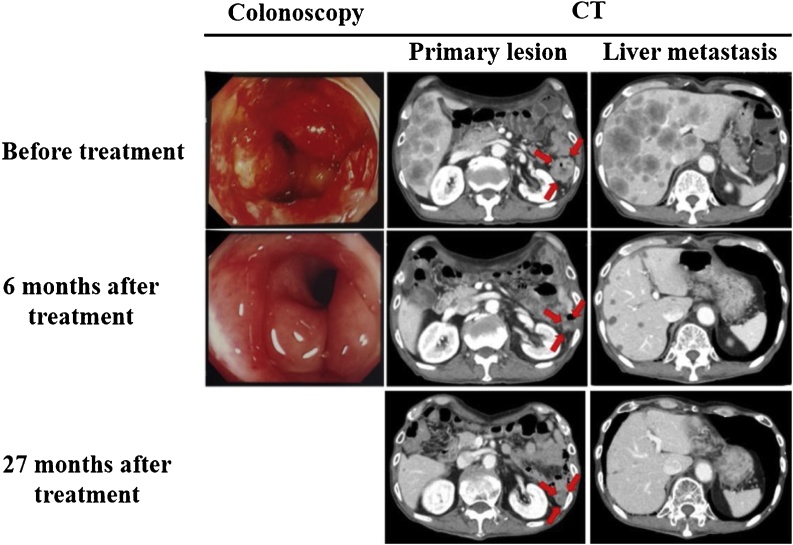
The occlusion of the descending colon improved, and no malignant findings were observed in the biopsy. CT scan also showed marked decrease in lesions, particularly, liver metastasis.

Abdominal contrast-enhanced computed tomography scan revealed advanced descending colon cancer and swelling of the paracolic lymph nodes. Numerous masses in the liver were found. No peritoneal seeding was observed (Fig. 1). Colonoscopy revealed circumferential type 2 lesions in the descending colon. Histopathological examination of the biopsy sample revealed highly-to-moderately differentiated wild-type KRAS adenocarcinoma.

**Fig. 2:**
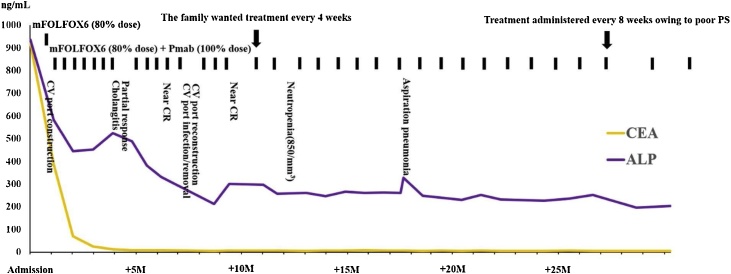
Clinical course.

Upon admission, after instituting fasting and parenteral nutrition and confirming improved large intestine obstruction symptoms, mFOLFOX6 + Pmab were administered. mFOLFOX6 (80% dose) was started 7 days later and mFOLFOX6(80% dose) + Pmab (100% dose) at 21 days. The treatment led to decreased tumor marker levels and enabled the patient to resume oral intake after five cycles completed. A follow-up computed tomography scan and colonoscopy performed 192 days after examination confirmed near CR (per Response Evaluation Criteria in Solid Tumors criteria). At 2 years and 8 months after the examination, he completed a total of 35 cycles and maintained the near CR status. The patient also developed mild cholangitis during that period, which was treated with antibiotics. Furthermore, a central venous port infection was observed; hence, the port was changed. No other serious adverse events were observed. The use of mFOLFOX6 + Pmab in this patient led to near CR that has been maintained without surgical treatment at the time of writing this report at 32 weeks after treatment (Fig. 2).

## 3 Discussion

According to the National Comprehensive Cancer Network guidelines, anti-cancer drugs selected for advanced, unresectable, and recurrent colorectal cancer can differ based on the indication for intensive therapy [3]. In general, BSC is the recommended option for elderly patients with advanced unresectable colon cancer who have risk factors, poor PS, and multiple distant metastases. Sargent et al. [4]. analyzed the actual PS and the results of first-line treatment against metastatic colorectal cancer and described the need for new approaches due to a significantly lower progression-free survival, overall survival (OS), and success rates in patients with PS 2 cancer compared to those with PS 0 or PS 1. They also reported high rates of adverse events and 60-day mortality among patients with grade 3 or higher.

PubMed was searched using the following keywords: ‘metastatic colon cancer,’ ‘elderly’, ‘poor performance status’, and ‘liver dysfunction’, which yielded a total of four case reports on elderly patients (age >65 years) with advanced colorectal cancer with liver metastasis and underwent chemotherapy with a PS of 2 or higher (Table 1) [[5], [6], [7], [8]].

**Table 1:** Literature review.

	Patient 1	Patient 2	Patient 3	Patient 4	Present case
Age (years), sex	72, M	71, F	74, F	67, F	80, M
ECOG performance status	3	3	3	2	3
Cancer site	Sigmoid colon	Sigmoid colon	Ascending colon	Ascending colon	Descending colon
Pathology	Adenocarcinoma	Adenocarcinoma	Neuroendocrine carcinoma	–	Adenocarcinoma
Metastatic site	Liver	Liver	Liver	Liver	Liver
Prior chemotherapy	Cmab Cmab + FOLFOX	HAI of 5-FU mFOLFOX + Bev	Cisplatin/Irinotecan	Irinotecan + Cmab	mFOLFOX6 mFOLFOX6 + Pmab
Reason for choice of chemotherapy	Absence of liver disorder	Improvement of PS with topical therapy	Poor PS	Poor PS	Poor PS Absence of liver disorder
Total bilirubin (mg/dL)	6.2	0.4	1.1	–	0.9
Aspartate transaminase (U/L)	258	51	79	–	86
Alanine transaminase (U/L)	98	22	28	–	75
Alkaline phosphatase (IU/L)	2,085	1,028	1,679	–	937
KRAS status	Wild	–	–	Wild	Wild
CEA (ng/mL)	894.9	1,408.8	–	976.4	921
Drop in bilirubin level	Yes	–	–	–	No
Drop in CEA level	Yes	–	–	Yes	Yes
Maximal toxicity, grade	Skin toxicity, G2	Hand-foot syndrome, G3	Neutropenia, G3	Diarrhea, G2	Rash, G1
Survival (months)	18.0	29.0	8.0	8.0	32.0

Abbreviations: M, male; F, female; ECOG, Eastern Cooperative Oncology Group; Cmab, cetuximab; HAI, hepatic arterial infusion; Bev, bevacizumab; Pmab, panitumumab; mFOLFOX6, modified 5-FU + leucovorin + oxaliplatin.

Decrease in bilirubin or CEA levels represents a 50% decrease in serum total bilirubin or CEA levels. The bar indicates that the measurement is not described in this paper.

Modified from: Elsoueidi R, Craig J, Mourad H, Richa E. Safety and efficacy of FOLFOX followed by cetuximab for metastatic colorectal cancer with severe liver dysfunction. J Natl Compr Canc Netw. 2014; 12:155–160. https://doi.org/10.6004/jnccn.2014.0016.

In one of the four cases, systematic therapy performed after hepatic arterial infusion resulted in improved liver function. In that case, the primary lesion was resected first; however, his PS became poor in the postoperative period. In two of the four cases, cetuximab was used (in combination), while the amount of cisplatin/irinotecan was reduced by 50% in one of the four cases, which was a case of primary small cell carcinoma in the large intestine.

This is the first case in which an elderly patient with poor PS and advanced unresectable colorectal cancer was treated with combination chemotherapy of Pmab. Chan et al. [9] studied 1013 young (<70 years) and elderly (≥70 years) patients with metastatic colon cancer with PS 0–1 administered with systemic therapy. The results of their analysis showed no significant difference in OS durations between the young and elderly.

Additionally, Naeim et al. [1] analyzed the combined use of capecitabine and bevacizumab treatment in frail (ECOG PS 2) or elderly patients with ECOG PS 1, and several patients with PS 2 achieved an overall response rate equivalent to those treated with fluorouracil (5-FU) + bevacizumab. This result also suggests the potential efficacy of using chemotherapy in elderly patients with poor PS.

Moreover, Fleming et al. [10] showed that combined therapy using a continuous infusion of 5-FU and leucovorin could be safely performed even in patients with jaundice, and liver function is also thought to not affect the pharmacokinetics of oxaliplatin. Moreover, the NCCN guidelines [3] recommend single administration of Pmab as the first-line treatment in patients with poor general condition; however, no studies on the onset of serious liver dysfunction were conducted based on this recommendation [11], leading to a conclusion that single administration of Pmab could also be a treatment option in patients who cannot be administered with other standard treatments due to liver dysfunction.

When planning the treatment for the present patient, the following information was considered. Based on the reports by Chan et al. [9], the systematic chemotherapy was expected to extend the OS in elderly patients. Naeim et al.’s [1] report suggested that performing systematic chemotherapy, even in cases of poor PS, may improve the OS and overall response rate. Fleming et al. [10] and Van Cutsem et al. [11] indicated that oxaliplatin, Pmab, or combined treatment of continuous 5-FU infusion and leucovorin can be used, even in patients with impaired liver function. For the initial administration, the dosage of mFOLFOX6 (80% dose) was reduced and the absence of major adverse events was confirmed. Then, we began a combined treatment with mFOLFOX6 (80% dose) + Pmab (100% dose). Because the patient progressed with no major side effects, the 35-cycle regimen was continued until his condition worsened to PS 4.

Although reports of using chemotherapy to treat advanced unresectable colorectal cancer in elderly patients or those with poor PS are limited, the information obtained in this case led us to conclude that systemic therapy was an option. Future accumulation of cases that use this therapy will lead to a comparison of the associated results with those of traditional therapies, such as BSC.

## 4 Conclusion

We encountered a case of near CR that was maintained long-term after using mFOLFOX6 (80% dose) + Pmab (100% dose) in an elderly patient with poor PS. The advanced unresectable colorectal cancer was accompanied by stricture, and multiple liver metastases were accompanied by elevated hepatobiliary system enzymes. Although BSC would typically have been chosen for this patient, chemotherapy was attempted, and long-term survival without requiring surgery was achieved. This suggests that advances in chemotherapy have made it possible to consider aggressive treatment as an option for severe advanced colorectal cancer that was managed with BSC in the past.

## Conflict of interest

We have nothing to declare in any categories.

## Sources of funding

This research does not receive any specific grant from funding agencies in the public, commercial, or not-for-profit sectors.

## Ethical approval

On Ethical Guidelines for Medical and Health Research Involving Human Subjects, case report is not classified in the research study in Japan.

## Consent

Written informed consent was obtained from the patient’s family for publication of this case report and accompanying images. The patient is currently in poor general condition due to pneumonia and cannot show consent. A copy of the written consent is available for review by the Editor-in-Chief of this journal on request”.

## Author contribution

Yoshiaki Kanemoto: conceptualization, validation, investigation, writing-original draft, writing-review & editing, visualization.

Giichiro Tsurita: conceptualization, methodology, writing-review & editing, supervision, project administration.

Tomohiro Kurokawa: conceptualization, methodology, validation, writing-review & editing, supervision.

Yuki Azuma: supervision.

Kentaro Yazawa: supervision.

Yoshinori Murakami: supervision.

## Registration of research studies

researchregistry4664.

## Guarantor

Giichiro Tsurita.

## Provenance and peer review

Not commissioned, externally peer-reviewed.
